# Lactational Exposure to Polybrominated Diphenyl Ethers and Its Relation to Social and Emotional Development among Toddlers

**DOI:** 10.1289/ehp.1205100

**Published:** 2012-07-19

**Authors:** Kate Hoffman, Margaret Adgent, Barbara Davis Goldman, Andreas Sjödin, Julie L. Daniels

**Affiliations:** 1 University of North Carolina Gillings School of Global Public Health, Chapel Hill, North Carolina, USA; 2 National Institute of Environmental Health Sciences, National Institutes of Health, Department of Health and Human Services, Research Triangle Park, North Carolina, USA; 3 Frank Porter Graham Child Development Institute and Department of Psychology, University of North Carolina, Chapel Hill, North Carolina, USA; 4 Centers for Disease Control and Prevention, National Center for Environmental Health, Division for Laboratory Sciences, Atlanta, Georgia, USA

**Keywords:** neurodevelopment, polybrominated diphenyl ethers (PBDEs), social and emotional development

## Abstract

Background: Polybrominated diphenyl ethers (PBDEs) have been widely used as flame retardants and are ubiquitous environmental contaminants. PBDEs have been linked to adverse neurodevelopment in animals and humans.

Objectives: We investigated the association between breast milk PBDE levels and social and emotional development in toddlers.

Methods: The Pregnancy Infection and Nutrition (PIN) and PIN Babies studies followed a cohort of North Carolina pregnant women and their children through 36 months of age. Breast milk samples obtained at 3 months postpartum were analyzed for PBDEs. The Infant–Toddler Social and Emotional Assessment (ITSEA) was completed by mothers when children were approximately 30 months of age (*n* = 222). We assessed the relationship between breast milk concentrations of five PBDE congeners—BDEs 28, 47, 99, 100, and 153—and children’s social and emotional development, adjusting for other factors.

Results: A small, imprecise, yet consistent positive association was apparent between BDEs 47, 99, and 100 and increased externalizing behaviors, specifically activity/impulsivity behaviors. Externalizing domain *T*-scores ranged from 30 to 87 with a mean of 47.8. Compared with those with BDE-47 concentrations below the median, adjusted externalizing behavior domain scores were 1.6 [95% confidence interval (CI): –1.2, 4.4] and 2.8 (95% CI –0.1, 5.7) points higher for children born to women with breast milk concentrations in the 3rd and 4th quartiles, respectively. PBDEs were not associated with other social and emotional developmental domains.

Conclusions: Our results, although imprecise, suggest a subtle association between early-life PBDE exposure and increased activity/impulsivity behaviors in early childhood. Confirmation of these results is needed in other longitudinal studies.

Polybrominated diphenyl ethers (PBDEs) have been widely used as flame retardants in a variety of household products including electronics, carpets, and polyurethane foams used to make furniture (Darnerud et al. 2001). Unlike reactive flame retardants, PBDEs are not chemically bound to products, increasing their potential to leach into and persist in the environment (Sjödin et al. 2003). Consequently, they have been detected in the environment, animals, and humans throughout the world (Darnerud et al. 2001; Hites 2004).

The primary sources of exposure to PBDEs for the general population are thought to be diet and dust in the home and workplace environments (Darnerud et al. 2001; Fraser et al. 2009; Johnson et al. 2010; Wu et al. 2007). For young children, PBDE exposures via the mother, both *in utero* and through nursing, contribute significantly to the overall body burden (Johnson-Restrepo and Kannan 2009; Toms et al. 2009). Exposure through breast milk is of particular concern as PBDEs are lipophilic and are concentrated in breast milk (Daniels et al. 2010). Additionally, the sensitivity of the developing brain during the prenatal period and early childhood raises concern about the impact of PBDEs on neurodevelopment, particularly because it has been demonstrated that young children have higher levels of PBDEs in their bodies than do adults (Lunder et al. 2010).

A large body of animal research has accumulated information that indicates adverse effects of prenatal and early-life exposures to PBDEs on neurodevelopment, including changes in spontaneous motor activity characterized by hyperactivity, decreased habituation, and disruption of learning and memory (Costa and Giordano 2007). Administered levels leading to adverse effects on neurodevelopment in animals are similar to exposure levels for infants (Costa and Giordano 2007). Data in humans remain sparse and conflicting. In a cohort of children from New York City, increasing levels of BDEs 47, 99, and 100 in umbilical cord blood were associated with significantly lower scores on tests of mental and psychomotor development at 12–36 months of age and of cognitive development at 48 and 72 months (*n* = 96–118, depending on year of assessment; Herbstman et al. 2010). In a study of 62 Dutch school-age children, prenatal serum concentrations of PBDEs were both positively and negatively associated with changes in motor skills (fine manipulative ability and coordination), attention, and behavior (Roze et al. 2009).

We assessed the association between exposure to PBDEs and early social and emotional development in children using PBDE concentrations in human milk as an index of exposure. Using data from the Pregnancy Infection and Nutrition (PIN) Babies Study we investigated the association prospectively in a well-characterized cohort of central North Carolina children 30 months of age.

## Methods

*Study population.* The PIN Study followed a cohort of central North Carolina women from early pregnancy through 12 months postpartum (PIN 2011). In the third phase of PIN, women were recruited from the University of North Carolina prenatal care clinic, and they delivered infants at University of North Carolina hospitals between 2001 and 2005 (*n* = 2009). The PIN Postpartum Study followed women through the first year postpartum (*n* = 689). The PIN Babies Study began in January 2004 to follow the children of women participating in the later years of the PIN study through 3 years of age (*n* = 585). By design, all children in the PIN Babies Study were singleton births free from major birth defects. This analysis was further limited to infants who were primarily breast-fed at least 3 months, to obtain a milk sample (*n* = 304). Women participating in the PIN Babies Study were more likely to be white, have higher educational attainment, and be older than mothers in the larger PIN cohort (Daniels et al. 2010; PIN 2011). Self-administered questionnaires, telephone interviews, and home visits were used to collect pregnancy and postpartum health and lifestyle information throughout the PIN studies. All study protocols were approved by the institutional review boards at the University of North Carolina–Chapel Hill and the Centers for Disease Control and Prevention, and all mothers provided informed consent.

*Sample collection.* Details of the breast milk sample collection were published previously (Daniels et al. 2010). Briefly, lactating women provided a morning milk sample at 3 months postpartum using the milk collection kit provided. Women stored the milk in their freezer until a research team picked up the sample during a home data collection visit later the same day. Samples were then stored at –80°C until analyzed.

*Sample analysis.* The Organic Analytical Toxicology Branch of the National Center for Environmental Health at the Centers for Disease Control and Prevention (Atlanta, GA) analyzed milk samples for nine PBDE congeners using previously described methods (Sjödin et al. 2004). The milk samples were added to diatomaceous earth packed in a solid-phase extraction cartridge (3 mL) and ^13^C-labeled internal standards were added. Target analytes and lipids were extracted using an automated modular solid-phase extraction system (Cambridge Isotope Laboratories, Andover, MA), which dried the sample onto the diatomaceous earth with pressurized nitrogen and eluted analytes and lipids with dichloromethane. Lipid content was determined gravimetrically, and the final analytical determination of PBDEs was performed by gas chromatography/isotope-dilution high-resolution mass spectrometry. Both wet weight and lipid normalized concentrations (reported as nanograms per gram lipid) were produced. PBDE values below the limit of detection (LOD) were assigned a concentration of the LOD divided by the square root of 2. For quality control, two quality control and two blank samples were added to each batch of 16 study samples. Quality assurance practices in the laboratory were regularly monitored (Sjödin et al. 2004).

Five PBDE congeners—BDEs 28, 47, 99, 100, and 153—were detected in > 91% of samples. We evaluated the level of these congeners, as well as their sum, in relation to developmental outcomes. The four PBDEs congeners infrequently detected [in < 70% of the PIN Babies sample; BDEs 66, 85, 154, and 183 (Daniels et al. 2010)] were not evaluated in relation to developmental outcomes.

*Social and emotional development assessment.* Mothers completed the Infant–Toddler Social and Emotional Assessment (ITSEA), of their child between 24 and 36 months of age (Carter and Briggs-Gowan 2006). The ITSEA is a validated measure of social and emotional development that uses a parent-report questionnaire to assess a wide array of social-emotional and behavioral problems and competencies. Parents rate a broad range of 166 behaviors that are part of typical development, but can be problematic if they occur too frequently or infrequently, as well as behaviors that occur infrequently and represent deviations from the normal course of development. The parent scores each item as 0 = not true/rarely, 1 = somewhat true/sometimes, 2 = very true/often. Mothers were not aware of the levels of PBDEs in their breast milk at the time the ITSEA was completed.

The ITSEA produces scores for four primary domains of social and emotional behavioral development for young children; each domain contains several subscales: externalizing domain (activity/impulsivity, aggression/defiance, peer aggression), internalizing domain (depression/withdrawal, general anxiety, separation distress, inhibition to novelty), dysregulation (problems with sleeping, problems with eating, negative emotionality, sensory sensitivity), and social-emotional competence domain (positive behaviors that include compliance, attention regulation, imitation and pretend play skills, mastery motivation, empathy, prosocial peer relations). Previous research demonstrates good test–retest reliability, criterion validity, and a well-supported factor structure in the ITSEA (Carter and Briggs-Gowan 2006; Carter et al. 2003).

For each of the four main behavioral domains, subscales are averaged and converted to age- and sex-specific *T*-scores that have a normalized mean of 50 and a standard deviation of 10. Higher scores in the externalizing, internalizing, and dysregulation domains indicate more behavior problems, whereas higher scores in the social-emotional competence domain indicate better behavior. Normative *T*-scores are not available for each of the 17 subscales that constitute the ITSEA domain scores; only the raw score percentile ranks are available for each subscale. The ITSEA subscales conventionally use the 10th percentile to indicate clinical concern (using normative data). Compared with the normative population, distributions in our sample were, on average, shifted toward slightly better behavior; thus, we dichotomized each subscale at the 20th percentile to include scores indicating possible problems that may characterize children at risk, relative to the norm in this sample, as well as those with more extreme scores that would typically raise significant concern. Although subscales may be less reliable than domain scores because they are based on fewer items (5–13), and subscales are not age- and sex-standardized for our population, they provide additional detail for specific, more common, behavior clusters.

Additional maternal demographic, psychosocial, and lifestyle covariates were collected through PIN and PIN Postpartum maternal interviews (PIN 2011). PIN Babies staff completed a modified version of the Home Observation for Measurement of the Environment (HOME) Inventory (Caldwell and Bradley 1984) during the 36-month interviews to obtain information about parenting styles and influences of the home environment on the child. The modified HOME included only variables that could be accessed through interview. Study staff were not aware of a child’s ITSEA scores or PBDE exposures through breast milk at the time the HOME was administered.

*Statistical analysis.* We examined the shape of the relationship between the milk concentrations of each of the five PBDE congeners and their sum, using both wet weight and lipid-adjusted PBDE levels, and the continuous domain *T*-scores using a locally weighted regression smoother (LOESS). Patterns of association were similar using both methods; results using lipid adjusted levels are reported herein. On the basis of the visual inspection of plots of each smoothed association, we created three categories for breast milk PBDE levels: below the 50th percentile; from the 50th to 75th percentiles; and above the 75th percentile. We used separate multivariate regression models to examine the association between each of the five most detected PBDEs and their sum and the four ITSEA domains. We used logistic regression models to assess the relationship between these five PBDEs and their sum and the 17 dichotomous subscale scores.

We adjusted models for child sex and age; household income; maternal age, race, and education; parity; prenatal tobacco use; omega-3 fatty acid levels; and duration of breast-feeding. We carefully examined the appropriate form (continuous or nominal) for each covariate in relation to the outcomes and present the categorization used in Table 1. These covariates were chosen based on our *a priori* expectation of their association with both PBDE milk concentrations and indicators of social and emotional problems and competence. We also considered including the HOME score in adjusted analyses, but did not because variability in the HOME was minimal within our population and generally explained by differences in income and educational attainment (Caldwell and Bradley 1984). All analyses were conducted in SAS version 9.2 (SAS Institute Inc., Cary, NC).

**Table 1:** Selected sample characteristics.

Characteristic	n (%) or mean ± SD	
Total population	222 (100.0)	
Sex
Male	119 (53.6)	
Female	103 (46.4)	
Household income
≤ $35,000	34 (15.3)	
> $35,000	188 (84.7)	
Maternal race
White	198 (89.2)	
Nonwhite	24 (10.8)	
Maternal education (years)
< 15	36 (16.2)	
≥ 16	186 (83.8)	
Maternal age (years)
≤ 25	31 (14.0)	
26–30	75 (33.8)	
31–35	82 (36.9)	
≥ 36	34 (15.3)	
Parity
0	117 (52.7)	
≥ 1	105 (47.3)	
Tobacco usea
Yes	9 (4.1)	
No	211 (95.9)	
Breast-feeding (months)a
< 10	78 (35.1)	
10–14	97 (43.7)	
≥ 15	43 (19.4)	
Child age (months)	30.3 ± 4.4	
Externalizing behavior problems	47.8 ± 8.8	
Internalizing behavior problems	45.1 ± 9.0	
Dysregulation	43.0 ± 10.1	
Social-emotional competencya	57.6 ± 10.7	
aMissing tobacco use during pregnancy n = 2; missing breast-feeding duration n = 4; missing social-emotional competency n = 1.			

## Results

Five-hundred-eighty-nine women participated in PIN Babies; 304 of these women reported exclusively or mostly breast-feeding at 3 months postpartum and provided breast milk samples. Of these mothers, 222 (67.1%) returned an ITSEA for their child. Most mothers in our study population were white and had completed ≥ 16 years of education at the time of the child’s birth (Table 1). By design, all women in our study were breast-feeding in the third month postpartum, and 63.1% (*n* = 140) reported breast-feeding for at least 10 months. Compared with women in the PIN Babies population as a whole, mothers in our study sample were more likely to be white, have higher educational attainment at the time of their child’s birth, and were more likely to continue breast-feeding their children for at least 10 months (data not shown).

BDE-99 was detected in 91.0% of breast milk samples, and BDEs 28, 47, 100, and 153 were all detected in at least 97.3% of samples (Table 2). The median concentrations of BDEs 28, 47, 99, 100, and 153 were 2.2, 28.7, 5.5, 5.3, and 5.6 ng/g lipid, respectively. As reported previously, levels of PBDEs in breast milk were slightly higher in the PIN Babies population than in other similarly timed U.S. samples (Daniels et al. 2010). Congener concentrations were highly correlated (*r* = 0.53–0.93).

**Table 2:** Lipid-adjusted concentrations (ng/g lipid) of commonly detected PBDEs in 222 breast milk samples collected at 3 months postpartum in 2004–2006.

PBDE congener	IUPAC number	Mean (median)	Range	Percent detected (LOD)
2,4,4’-Tribromodiphenyl ether	BDE-28	3.4 (2.2)	ND–49.6	97.3 (0.3)
2,2’,4,4’-Tetrabromodiphenyl ether	BDE-47	50.2 (28.7)	4.0–1430.0	100.0 (1.3)
2,2’,4,4’,5-Pentabromodiphenyl ether	BDE-99	10.4 (5.5)	ND–299.0	91.0 (1.1)
2,2’,4,4’,6-Pentabromodiphenyl ether	BDE-100	10.6 (5.3)	ND–188.0	99.1 (0.3)
2,2’,4,4’,5,5’-Hexabromodiphenyl ether	BDE-153	14.7 (5.6)	ND–229.0	98.7 (0.3)
IUPAC, International Union of Pure and Applied Chemistry. ND (nondetectable) values are treated as the LOD divided by the square root of 2 in the calculation of the mean.								

In comparison to the social and emotional domains measured by the ITSEA in the normative population (mean normative *T* = 50), children in our sample generally had lower scores on assessments of externalizing behavior problems, internalizing behavior problems, and dysregulation (indicating fewer behavior problems; age- and sex-adjusted domain *T*-scores: 47.8, 45.1, and 43.0, respectively) (Table 1). For the competency domain, where higher scores indicate better performance, the adjusted mean *T*-score was 57.6 in our sample.

**Figure 1:**
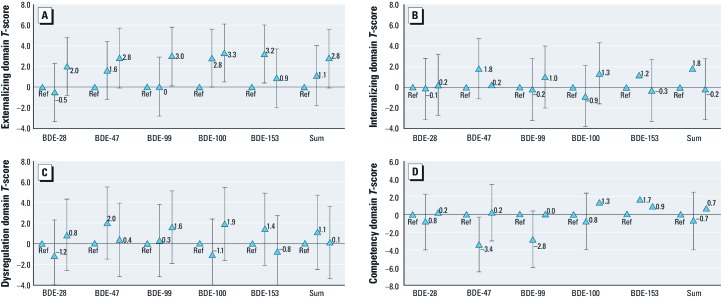
Associations (adjusted betas) between each PDBE congener and ITSEA domain T-scores. PBDE congener concentrations in breast milk modeled as below the median (referent group; Ref), the 50th–75th percentile, and above the 75th percentile. Triangles indicate the β estimate and error bars indicate the 95% CI. (*A*) Externalizing behavior problems domain. (*B*) Internalizing behavior problems domain. (*C*) Dysregulation domain. (*D*) Social-emotional competency domain. Lower scores indicate poorer performance for social-emotional competency; higher scores indicate poorer performance for the other three domains.

Higher levels of BDEs 47, 99, 100 or the sum of the five congeners in breast milk were consistently associated with increased externalizing behavior problems in the child [Figure 1; see also Supplemental Material, Table S1 for numeric data (http://dx.doi.org/10.1289.ehp.1205100)]. Children in the 4th quartile of exposure to BDE-47, estimated from the levels of the PBDEs in breast milk, scored 2.8 points higher in the externalizing behavior domain [95% confidence interval (CI): –0.1, 5.7], suggesting increased externalizing behavioral problems. Similarly, for BDE-99, externalizing behavior scores were 3.0 points higher for children with exposures in the highest (4th) quartile (95% CI: 0.1, 5.8) compared with those with exposures below the median. Associations between levels of BDEs 28, 47, 99, 100, 153 or the sum of the five congeners in breast milk and internalizing, dysregulation, and competency domains were generally small, inconsistent across congeners, and imprecise (Figure 1; see also Supplemental Material, Table S1). Results were similar when we compared the 3rd and 4th quartiles to the 1st quartile, instead of below the median (data not shown).

**Figure 2:**
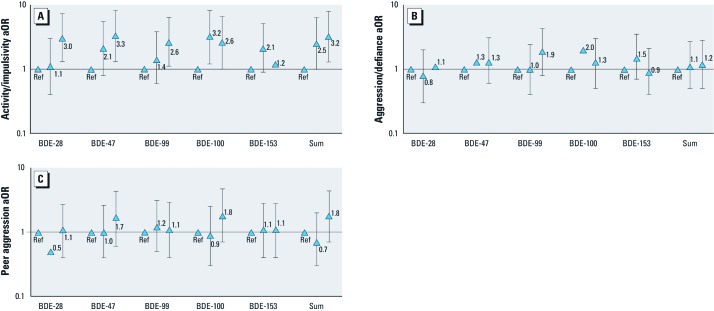
Associations (adjusted odds ratios; aORs) between each PDBE congener and the three externalizing subscales. PBDE congener concentrations in breast milk modeled as below the median (referent group; Ref), the 50th–75th percentile, and above the 75th percentile. Triangles indicate the aOR estimate and error bars indicate the 95% CI. (*A*) Activity/impulsivity. (*B*) Aggression/defiance. (*C*) Peer aggression.

Within the externalizing behavior domain subscales, the probability of high scores on the activity/impulsivity subscale increased with milk concentrations of BDEs 28, 47, 99, 100, and the sum of the five congeners [Figure 2; see Supplemental Material, Table S2 for numeric data for all subscales (http://dx.doi.org/10.1289.ehp.1205100)]. For example, children whose mothers had levels of breast milk BDE-47 at the 3rd and 4th quartiles were 2.1 (95% CI: 0.8, 5.4) and 3.3 (95% CI: 1.3, 8.2) times more likely to have activity/impulsivity subscale scores in the top 20% of our sample compared with children whose mothers had milk levels of these BDEs below the median. We did not observe obvious patterns of association for the other subscales of the externalizing domain (aggression/defiance and peer aggression) and these PBDE concentrations (Figure 2; see Supplemental Material, Table S2).

Outside of externalizing behaviors, associations between PBDEs and individual subscale scores generally reflected the patterns observed for the domain score. Results were largely null and associations were inconsistent across both the concentration (used to assess exposure) gradient and congeners [see Supplemental Material, Table S2 (http://dx.doi.org/10.1289.ehp.1205100)]. One possible exception was empathy behaviors, which were worse in higher quartiles of concentrations for BDEs 28, 47, and 99. Additionally, there was some evidence of increased general anxiety with increasing concentrations of BDEs 28, 99, and 100 and the sum of the five congeners; however, results were imprecisely estimated (see Supplemental Material, Table S2).

## Discussion

Our results suggest a modest but imprecisely estimated association between early-life exposures to certain PBDEs through breast milk and increased externalizing behaviors in early childhood, primarily driven by activity/impulsivity behaviors. Children who had high activity/impulsivity subscale scores were rated by their parents as very active, fidgety, having trouble sitting still, or having difficulty inhibiting their actions (Carter and Briggs-Gowan 2006). Although these children were young and the ITSEA is not intended to be used as a diagnostic tool by itself, young children who are later diagnosed with attention deficit/hyperactivity disorder (ADHD) would also be expected to have had high activity/impulsivity subscale scores on the ITSEA (Carter and Briggs-Gowan 2006). Most other aspects of early social and emotional behavior that we assessed were not associated with postnatal breast milk PBDE concentrations.

Our results are consistent with toxicologic data indicating that PBDE exposure impacts neurodevelopment [reviewed by Costa and Giordano (2007)]. For example, both prenatal and postnatal PBDE exposures have been linked to adverse neurodevelopmental outcomes including increased hyperactivity in rodents (Branchi et al. 2002, 2005; Gee and Moser 2008; Kuriyama et al. 2005). Epidemiologic data are sparse; to our knowledge, these results are the first to prospectively assess BDEs 28, 47, 99, 100, and 153 levels in breast milk and social and emotional behavioral development. In contrast with our results, the only other published study of PBDEs and human behavior reported better behavior in children 5–6 years of age with higher prenatal exposure to BDE-100 (Roze et al. 2009). Our results may differ from those of Roze et al. (2009) because of methodological differences: Our study sample was larger, concentrations were measured at different time points (prenatal vs. postnatal) and in different matrices (maternal serum vs. breast milk) and development was assessed at a younger age using a different instrument. Herbstman et al. (2010) also reported detrimental developmental impacts associated with umbilical cord levels of BDEs 47, 99, and 100, but focused on prenatal versus postnatal exposure and cognitive versus behavioral development.

Concentrations of PBDE congeners are generally highly correlated, a finding that was also observed in this study (*r* = 0.53–0.93). Such correlations raise questions about the interpretation of the analysis on any one congener. Toxicologic experiments suggest that BDE-99 may be a more potent congener (Viberg et al. 2003), but one of the few epidemiologic studies reported stronger associations between BDE-100 and cognitive development (Herbstman et al. 2010). Although we observed associations across several congeners, we cannot rule out the possibility that our results are driven by a single congener. Using an additive summary measure of total PBDE levels in models produced similar results to models with individual congeners; this result was anticipated because PBDE levels in milk were highly correlated in this population. In addition, our inferences are limited by our reliance on a single postnatal measure of PBDEs. The infants in this study were primarily breast-fed through the first several months of life, conferring continual exposure to PBDEs during a time of accelerated brain growth. However, we are unable to speculate how PBDE exposure *in utero* may affect fetal brain development from these data. Although small studies have reported some correlation between PBDE levels in maternal serum, umbilical cord blood, and breast milk (Guvenius et al. 2003; Kim et al. 2012; Mazdai et al. 2003; Needham et al. 2010; Schecter et al. 2010), partitioning between breast milk and maternal serum varies by congener. For example, BDE-47 was recently reported as higher in breast milk than maternal serum, whereas BDE-153 partitioned equally between milk and serum (Schecter et al. 2010). Because we measured PBDEs only in breast milk, we were unable to assess the cumulative or independent effects of *in utero* and postnatal exposures. As others report relations between PBDEs and developmental outcomes, the exposure matrix should be considered and the correlation between such matrices should be evaluated when possible.

The PIN Babies Study cohort is well characterized and larger than other epidemiologic cohorts to date, which allowed for careful adjustment of important covariates. However, the ability to detect sex-specific associations was limited by the size of the cohort. Sex may be an important factor in PBDE neurotoxicity, particularly because endocrine disruption is hypothesized to play a role in the potential neurotoxicity of PBDEs. The sample size may also have limited our ability to detect more subtle differences in social and emotional behavior (e.g., anxiety and empathy) with increasing concentrations.

The children in our study sample population generally were reported by their mothers to be performing well on the ITSEA, suggesting appropriate social and emotional development at an average of 30 months of age. However, our assessment of social and emotional development relied on maternal report. Use of data from multiple sources, including clinical assessments of the child and report of behavior in other settings (i.e., school), may provide additional insights into the impact of PBDE exposures in early life on neurodevelopment. Increases in externalizing behaviors associated with higher exposures were generally small, and the vast majority of children in our population, even those born to mothers with the highest concentrations of PBDEs in their breast milk, had social and emotional development scores within the normal range. Still, subtle difficulties in self-regulation may be related to childhood and adolescent mental health and behavioral problems (Shonkoff and Phillips 2000). Our focus on early childhood may underestimate the potential for the association between PBDEs and behavior later in development, as animal toxicology models demonstrate that the neurodevelopmental impact of early-life PBDE exposures may worsen or become more apparent with age (Viberg et al. 2003).

## Conclusions

Our results suggest an association between early-life exposures to PBDEs via breast milk and increased externalizing behaviors in early childhood, particularly for activity/impulsivity behaviors. Given the widespread exposure to PBDEs, confirmation of our results is needed in other longitudinal studies.

## Supplemental Material

(279 KB) PDF
