# Supplementary material for: Lactational Exposure to Polybrominated Diphenyl Ethers and Its Relation to Social and Emotional Development among Toddlers
Source: Environ Health Perspect. 2012 Jul 19;120(10):1438–42. doi: 10.1289/ehp.1205100 (PMC3491946; doi:10.1289/ehp.1205100)
Supplement: (279 KB) PDF [file ehp.1205100.s001.pdf]

Supplemental Material

Title

Lactational Exposure to Polybrominated Diphenyl Ethers and its Relation to Social and Emotional Development among Toddlers

Authors

Kate Hoffman
Margaret Adgent
Barbara Davis Goldman
Andreas Sjödin
Julie L. Daniels

Contents

Supplemental Material, Table S1..Page 2

Associations between levels of PBDE congeners in breast milk and ITSEA domains

Supplemental Material, Table S2..Page 3-5

Associations between levels of PBDE congeners in breast milk and subscales of behavior

Supplemental Material, Table S1: Adjusted* associations (Beta) between levels of PBDE congeners in breast milk and ITSEA domains (exposure categorized as below the median, the 50th to the 75th percentile, and above the 75th percentile of congener levels in breast milk).

Domain	BDE-28			BDE-47			SUM		
	below the median <2.0 ng/g lipid	50th to the 75th percentile 2.0-3.7 ng/g lipid	above the 75th percentile >3.7 ng/g lipid	below the median <27.7 ng/g lipid	50th to the 75th percentile 27.7-54.2 ng/g lipid	above the 75th percentile >54.2 ng/g lipid	below the median <5.2 ng/g lipid	50th to the 75th percentile 5.2-11.2 ng/g lipid	above the 75th percentile >11.2 ng/g lipid
**Externalizing**	Reference	-0.5 (-3.3, 2.3)	2.0 (-0.8, 4.8)	Reference	1.6 (-1.2, 4.4)	2.8 (-0.1, 5.7)	Reference	0.0 (-2.8, 2.9)	3.0 (0.1, 5.8)
**Internalizing**	Reference	-0.1 (-3.1, 2.8)	0.2 (-2.7, 3.2)	Reference	1.8 (-1.1, 4.7)	0.2 (-2.8, 3.2)	Reference	-0.2 (-3.2, 2.8)	1.0 (-2.0, 4.0)
**Dysregulation**	Reference	-1.2 (-4.7, 2.3)	0.8 (-2.6, 4.3)	Reference	2.0 (-1.5, 5.5)	0.4 (-3.2, 3.9)	Reference	0.3 (-3.2, 3.8)	1.6 (-1.9, 5.1)
**Competency**	Reference	-0.8 (-4.0, 2.3)	0.2 (-3.0, 3.3)	Reference	-3.4 (-6.5, -0.3)	0.2 (-3.0, 3.4)	Reference	-2.8 (-6.0, 0.4)	0.0 (-3.2, 3.2)

* Adjusted for gender (male vs. female), child age (continuous), household income (<=$35,000 vs. >$35,000), omega 3 fatty acid levels (continuous), parity (0 vs. 1+), mother's age (<=25 Years, 26-30 Years, 31-35 Years, >=36 Years), mother's race (white vs. non-white), breastfeeding duration (<10 months, 10-14 months, >=15 months), tobacco use during pregnancy (yes vs. no)

Supplemental Material, Table S1 (continued): Adjusted* associations (Beta) between levels of PBDE congeners in breast milk and ITSEA domains (exposure categorized as below the median, the 50th to the 75th percentile, and above the 75th percentile of congener levels in breast milk).

Domain	BDE-100			BDE-153			SUM		
	below the median <5.2ng/g lipid	50th to the 75th percentile 5.2-10.4 ng/g lipid	above the 75th percentile >10.4 ng/g lipid	below the median <5.6 ng/g lipid	50th to the 75th percentile 5.6-13.5 ng/g lipid	above the 75th percentile >13.5 ng/g lipid	below the median <51.2 ng/g lipid	50th to the 75th percentile 51.2-102.2 ng/g lipid	above the 75th percentile >102.2 ng/g lipid
**Externalizing**	Reference	2.8 (0.0, 5.6)	3.3 (0.5, 6.1)	Reference	3.2 (0.4, 6.0)	0.9 (-2.0, 3.7)	Reference	1.1 (-1.8, 4.0)	2.8 (-0.1, 5.6)
**Internalizing**	Reference	-0.9 (-3.8, 2.1)	1.3 (-1.6, 4.3)	Reference	1.2 (-1.7, 4.2)	-0.3 (-3.3, 2.7)	Reference	1.8 (-1.2, 4.8)	-0.2 (-3.1, 2.8)
**Dysregulation**	Reference	-1.1 (-4.6, 2.4)	1.9 (-1.6, 5.4)	Reference	1.4 (-2.1, 4.9)	-0.8 (-4.3, 2.7)	Reference	1.1 (-2.5, 4.7)	0.1 (-3.4, 3.6)
**Competency**	Reference	-0.8 (-4.0, 2.4)	1.3 (-2.0, 4.5)	Reference	1.7 (-1.5, 4.8)	0.9 (-2.3, 4.1)	Reference	-0.7 (-4.0, 2.5)	0.7 (-2.4, 3.9)

* Adjusted for gender (male vs. female), child age (continuous), household income (<=$35,000 vs. >$35,000), omega 3 fatty acid levels (continuous), parity (0 vs. 1+), mother's age (<=25 Years, 26-30 Years, 31-35 Years, >=36 Years), mother's race (white vs. non-white), breastfeeding duration (<10 months, 10-14 months, >=15 months), tobacco use during pregnancy (yes vs. no)

Supplemental Material, Table S2: Adjusted* associations (aOR) between levels of PBDE congeners in breast milk and subscales of behavior (exposure categorized as below the median, the 50th to the 75th percentile, and above the 75th percentile of congener levels in breast milk).

	Cut point for behavior problems (20th %ile in study population)	Mean ± SD**	BDE-28			BDE-47		
			below the median <2.0 ng/g lipid	50th to the 75th percentile 2.0-3.7 ng/g lipid	above the 75th percentile >3.7 ng/g lipid	below the median <27.7 ng/g lipid	50th to the 75th percentile 27.7-54.2 ng/g lipid	above the 75th percentile >54.2 ng/g lipid
**Activity/ Impulsivity**	**>=1.0**	**0.6 ± 0.7**	Reference	1.1 (0.4, 3.0)	3.0 (1.3, 7.3)	Reference	2.1 (0.8, 5.4)	3.3 (1.3, 8.2)
**Aggression/ Defiance**	**>0.7**	**0.5 ± 0.3**	Reference	0.8 (0.3, 2.0)	1.1 (0.5, 2.5)	Reference	1.3 (0.6, 3.0)	1.3 (0.6, 3.1)
**Peer Aggression**	**>0.3**	**0.1 ± 0.2**	Reference	0.5 (0.2, 1.3)	1.1 (0.4, 2.7)	Reference	1.0 (0.4, 2.6)	1.7 (0.6, 4.3)
**General Anxiety**	**>0.4**	**0.2 ± 0.2**	Reference	1.3 (0.6, 3.2)	1.5 (0.6, 3.5)	Reference	1.0 (0.4, 2.4)	1.2 (0.5, 3.0)
**Depression/ Withdrawal**	**>0.1**	**0.1 ± 0.1**	Reference	0.6 (0.2, 1.3)	0.8 (0.4, 1.7)	Reference	0.9 (0.4, 1.9)	1.0 (0.4, 2.2)
**Inhibition to Novelty**	**>1.2**	**0.8 ± 0.4**	Reference	0.9 (0.4, 2.2)	0.6 (0.3, 1.5)	Reference	1.6 (0.7, 3.4)	0.7 (0.3, 1.9)
**Separation Distress**	**>1.0**	**0.7 ± 0.3**	Reference	1.0 (0.4, 2.3)	1.4 (0.6, 3.1)	Reference	1.5 (0.7, 3.4)	1.2 (0.5, 2.9)
**Eating**	**>0.8**	**0.5 ± 0.3**	Reference	1.2 (0.5, 3.1)	0.5 (0.1, 1.4)	Reference	1.4 (0.5, 3.5)	0.4 (0.1, 1.4)
**Negative Emotionality**	**>0.7**	**0.5 ± 0.3**	Reference	1.3 (0.6, 2.8)	1.3 (0.6, 3.0)	Reference	1.5 (0.7, 3.3)	1.2 (0.5, 2.9)
**Sensory Sensitivity**	**>0.5**	**0.3 ± 0.3**	Reference	1.0 (0.4, 2.5)	1.0 (0.4, 2.7)	Reference	3.0 (1.2, 7.3)	1.1 (0.4, 3.4)
**Sleep**	**>0.8**	**0.4 ± 0.5**	Reference	0.6 (0.2, 1.7)	1.9 (0.9, 4.4)	Reference	0.9 (0.4, 2.3)	2.0 (0.9, 4.7)
**Attention**	**<1.2**	**1.6 ± 0.3**	Reference	1.3 (0.5, 3.6)	1.1 (0.4, 3.0)	Reference	1.6 (0.6, 4.4)	1.0 (0.3, 2.7)
**Compliance**	**<1.1**	**1.4 ± 0.3**	Reference	0.8 (0.4, 1.9)	1.2 (0.5, 2.6)	Reference	1.2 (0.5, 2.7)	1.6 (0.7, 3.5)
**Empathy**	**<1.0**	**1.4 ± 0.4**	Reference	1.4 (0.5, 3.6)	2.0 (0.8, 5.0)	Reference	1.9 (0.7, 4.8)	2.3 (0.9, 6.1)
**Initiation/ Play**	**<1.3**	**1.7 ± 0.3**	Reference	1.5 (0.6, 3.9)	1.1 (0.4, 3.2)	Reference	2.0 (0.8, 5.3)	1.2 (0.4, 3.6)
**Mastery Motivation**	**<1.5**	**1.7 ± 0.3**	Reference	1.7 (0.7, 3.9)	1.9 (0.8, 4.3)	Reference	1.5 (0.6, 3.3)	1.4 (0.6, 3.3)
**Peer Relatedness**	**<0.8**	**1.4 ± 0.4**	Reference	0.7 (0.2, 2.1)	1.9 (0.7, 4.8)	Reference	1.2 (0.4, 3.4)	1.6 (0.6, 4.2)

* Adjusted for gender (male vs. female), child age (continuous), household income (<=$35,000 vs. >$35,000), omega 3 fatty acid levels (continuous), parity (0 vs. 1+), mother's age (<=25 Years, 26-30 Years, 31-35 Years, >=36 Years), mother's race (white vs. non-white), breastfeeding duration (<10 months, 10-14 months, >=15 months), tobacco use during pregnancy (yes vs. no)

**Standard Deviation

Supplemental Material, Table S2 (Continued): Adjusted* associations (aOR) between levels of PBDE congeners in breast milk and subscales of behavior (exposure categorized as below the median, the 50th to the 75th percentile, and above the 75th percentile of congener levels in breast milk).

	BDE-99			BDE-100			BDE-153		
	below the median <5.2 ng/g lipid	50th to the 75th percentile 5.2-11.2 ng/g lipid	above the 75th percentile >11.2 ng/g lipid	below the median <5.2ng/g lipid	50th to the 75th percentile 5.2-10.4 ng/g lipid	above the 75th percentile >10.4 ng/g lipid	below the median <5.6 ng/g lipid	50th to the 75th percentile 5.6-13.5 ng/g lipid	above the 75th percentile >13.5 ng/g lipid
**Activity/ Impulsivity**	Reference	1.4 (0.6, 3.8)	2.6 (1.1, 6.3)	Reference	3.2 (1.2, 8.0)	2.6 (1.0, 6.5)	Reference	2.1 (0.9, 5.1)	1.2 (0.5, 3.0)
**Aggression/ Defiance**	Reference	1.0 (0.4, 2.4)	1.9 (0.8, 4.3)	Reference	2.0 (0.8, 4.6)	1.3 (0.5, 3.0)	Reference	1.5 (0.7, 3.5)	0.9 (0.4, 2.1)
**Peer Aggression**	Reference	1.2 (0.5, 3.1)	1.1 (0.4, 2.9)	Reference	0.9 (0.3, 2.5)	1.8 (0.7, 4.7)	Reference	1.1 (0.4, 2.8)	1.1 (0.4, 2.8)
**General Anxiety**	Reference	1.3 (0.6, 3.2)	1.7 (0.7, 4.0)	Reference	0.7 (0.3, 1.8)	2.1 (0.9, 4.7)	Reference	0.5 (0.2, 1.3)	1.7 (0.8, 3.9)
**Depression/ Withdrawal**	Reference	0.9 (0.4, 2.0)	1.2 (0.6, 2.7)	Reference	0.5 (0.2, 1.2)	0.6 (0.3, 1.4)	Reference	0.9 (0.4, 1.9)	0.6 (0.3, 1.3)
**Inhibition to Novelty**	Reference	0.9 (0.4, 2.2)	0.6 (0.2, 1.5)	Reference	0.7 (0.3, 1.6)	0.6 (0.3, 1.5)	Reference	1.3 (0.6, 3.0)	0.8 (0.3, 1.8)
**Separation Distress**	Reference	1.2 (0.5, 2.8)	1.5 (0.6, 3.4)	Reference	1.4 (0.6, 3.3)	1.7 (0.7, 3.8)	Reference	1.7 (0.8, 3.8)	0.6 (0.2, 1.4)
**Eating**	Reference	0.9 (0.3, 2.3)	0.4 (0.1, 1.2)	Reference	0.3 (0.1, 1.1)	0.4 (0.2, 1.2)	Reference	0.6 (0.2, 1.6)	0.3 (0.1, 1.1)
**Negative Emotionality**	Reference	1.6 (0.7, 3.6)	2.2 (1.0, 4.9)	Reference	1.5 (0.7, 3.3)	1.2 (0.5, 2.8)	Reference	2.0 (0.9, 4.5)	1.3 (0.6, 2.9)
**Sensory Sensitivity**	Reference	1.8 (0.7, 4.6)	1.0 (0.4, 2.8)	Reference	1.4 (0.6, 3.5)	0.9 (0.3, 2.4)	Reference	1.1 (0.5, 2.9)	0.7 (0.3, 1.9)
**Sleep**	Reference	0.6 (0.2, 1.6)	1.5 (0.7, 3.4)	Reference	0.8 (0.3, 2.1)	2.2 (0.9, 5.0)	Reference	1.2 (0.5, 2.9)	1.0 (0.4, 2.5)
**Attention**	Reference	1.6 (0.6, 4.3)	1.0 (0.3, 2.8)	Reference	2.1 (0.8, 5.6)	0.6 (0.2, 1.8)	Reference	2.4 (0.9, 6.4)	0.7 (0.2, 2.2)
**Compliance**	Reference	1.6 (0.7, 3.7)	1.2 (0.5, 2.8)	Reference	1.8 (0.8, 4.2)	1.3 (0.5, 3.0)	Reference	1.4 (0.6, 3.1)	0.7 (0.3, 1.6)
**Empathy**	Reference	3.2 (1.2, 8.5)	2.1 (0.8, 5.7)	Reference	1.6 (0.6, 4.2)	1.3 (0.5, 3.4)	Reference	0.5 (0.2, 1.5)	1.3 (0.5, 3.1)
**Initiation/ Play**	Reference	2.8 (1.7, 8.0)	1.5 (0.5, 4.3)	Reference	1.8 (0.7, 4.6)	0.8 (0.3, 2.4)	Reference	1.0 (0.4, 2.7)	0.8 (0.3, 2.3)
**Mastery Motivation**	Reference	1.5 (0.6, 3.6)	2.1 (0.9, 4.8)	Reference	0.6 (0.3, 1.5)	1.1 (0.5, 2.5)	Reference	1.4 (0.6, 3.3)	0.8 (0.3, 1.9)
**Peer Relatedness**	Reference	1.6 (0.6, 4.3)	1.3 (0.5, 3.6)	Reference	1.2 (0.4, 3.2)	1.1 (0.4, 3.0)	Reference	1.1 (0.4, 2.9)	0.8 (0.3, 2.3)

* Adjusted for gender (male vs. female), child age (continuous), household income (<=$35,000 vs. >$35,000), omega 3 fatty acid levels (continuous), parity (0 vs. 1+), mother's age (<=25 Years, 26-30 Years, 31-35 Years, >=36 Years), mother's race (white vs. non-white), breastfeeding duration (<10 months, 10-14 months, >=15 months), tobacco use during pregnancy (yes vs. no)

Supplemental Material, Table 2 (Continued): Adjusted* associations (aOR) between levels of PBDE congeners in breast milk and subscales of behavior (exposure categorized as below the median, the 50th to the 75th percentile, and above the 75th percentile of congener levels in breast milk).

	SUM PBDE		
	**below the median <51.2 ng/g lipid**	**50th to the 75th percentile 51.2-102.2 ng/g lipid**	**above the 75th percentile >102.2 ng/g lipid**
**Activity/ Impulsivity**	Reference	2.5 (1.0, 6.3)	3.2 (1.3, 7.8)
**Aggression/ Defiance**	Reference	1.1 (0.5, 2.7)	1.2 (0.5, 2.8)
**Peer Aggression**	Reference	0.7 (0.3, 2.0)	1.8 (0.7, 4.4)
**General Anxiety**	Reference	1.5 (0.6, 3.6)	1.7 (0.7, 4.0)
**Depression/ Withdrawal**	Reference	0.9 (0.4, 1.9)	0.6 (0.3, 1.3)
**Inhibition to Novelty**	Reference	1.4 (0.6, 3.2)	0.5 (0.2, 1.3)
**Separation Distress**	Reference	1.3 (0.6, 2.9)	1.1 (0.5, 2.5)
**Eating**	Reference	0.8 (0.3, 2.3)	0.6 (0.2, 1.7)
**Negative Emotionality**	Reference	2.2 (1.0, 5.0)	1.2 (0.5, 2.9)
**Sensory Sensitivity**	Reference	2.2 (0.9, 5.5)	0.8 (0.3, 2.3)
**Sleep**	Reference	0.7 (0.3, 1.8)	1.6 (0.7, 3.7)
**Attention**	Reference	3.0 (1.1, 8.5)	0.6 (0.2, 1.8)
**Compliance**	Reference	1.2 (0.5, 2.9)	1.5 (0.5, 3.3)
**Empathy**	Reference	1.0 (0.4, 2.7)	1.5 (0.6, 3.7)
**Initiation/ Play**	Reference	2.0 (0.8, 5.4)	0.9 (0.3, 2.7)
**Mastery Motivation**	Reference	1.3 (0.6, 2.9)	1.1 (0.5, 2.5)
**Peer Relatedness**	Reference	0.4 (0.1, 1.4)	1.5 (0.6, 3.8)

* Adjusted for gender (male vs. female), child age (continuous), household income (<=$35,000 vs. >$35,000), omega 3 fatty acid levels (continuous), parity (0 vs. 1+), mother's age (<=25 Years, 26-30 Years, 31-35 Years, >=36 Years), mother's race (white vs. non-white), breastfeeding duration (<10 months, 10-14 months, >=15 months), tobacco use during pregnancy (yes vs. no)
